# A Network-DEA model to evaluate the impact of quality and access on hospital performance

**DOI:** 10.1007/s10479-023-05362-x

**Published:** 2023-05-10

**Authors:** G. P. Afonso, D. C. Ferreira, J. R. Figueira

**Affiliations:** 1 CERIS, Instituto Superior Técnico, Universidade de Lisboa, Avenida Rovisco Pais, 1, 1049-001 Lisbon, Portugal; 2 CEG-IST, Instituto Superior Técnico, Universidade de Lisboa, Avenida Rovisco Pais, 1, 1049-001 Lisbon, Portugal

**Keywords:** Network Data Envelopment Analysis, Efficiency, Quality, Access, Trade-offs

## Abstract

The relationship between efficiency, quality, and access in healthcare is far from being well defined. In particular, there is no consensus on whether there is a trade-off between hospital performance and its social dimensions, such as the care appropriateness, safety, and access to proper health care. This study proposes a new approach based on the Network Data Envelopment Analysis (NDEA) to evaluate the existence of potential trade-offs between efficiency, quality, and access. The aim is to contribute for the heated debate around this topic with a novel approach. The suggested methodology combines a NDEA model with the weak disposability of outputs to handle with undesirable outputs related to the poor quality of care or the lack of access to appropriate and safe care. This combination results in a more realistic approach that has not yet been used to investigate this topic. We utilised data of the Portuguese National Health Service from 2016 to 2019, with four models and nineteen variables selected to quantify the efficiency, quality, and access to public hospital care in Portugal. A baseline efficiency score was calculated and compared with the performance scores obtained under two hypothetical scenarios to quantify the impact of each quality/access-related dimension on efficiency. The first scenario considers that each variable, individually, is at its best situation (for example, absence of septicaemia cases), and the second one, at its worst (e.g., all seen inpatients had a septicaemia case). The findings suggest that there might exist meaningful trade-offs between efficiency, quality, and access. Most variables exhibited a considerable and negative impact on the overall hospital efficiency. That is, we may expect a trade-off between efficiency and quality/access.

## Introduction

Measuring the efficiency in the healthcare sector has been brought to attention due to the high pressure of private providers as well as governments to achieve good quality care with the least expenditure possible. Over the last years, several authors, including Mitropoulos et al. (2013b) and Pereira et al. (2021), have been reinforcing the importance of measuring the efficiency of healthcare systems due to their high weight in the national budget as well as to meet financial objectives of providers to maintain economic viability.

However, as stated by Varabyova et al. (2017), efficiency cannot be the only focus in the healthcare sector. Quality and availability of resources are other two critical factors that impact the functioning of any National Health Service (NHS). As coined by Ferreira et al. (2020), these are the main social dimensions of hospital care, so we refer to both quality and access as social dimensions henceforward. Nevertheless, the impact that quality and access may have on performance is unclear (Ferreira et al., 2020; Kittelsen et al., 2015; Varabyova et al., 2017). There is no assurance that improving efficiency will reduce quality or access, neither the contrariwise case (Chatfield, 2014; Gorgemans et al., 2018; Ferreira & Marques, 2019).

Furthermore, over the last years, some authors have reached opposite conclusions concerning this issue. On the one hand, several studies concluded that it is possible to improve efficiency without jeopardising quality and access. This is the case of van Ineveld et al. (2016), Onder et al. (2021) and Khushalani and Ozcan (2017), according to whom it is possible to improve simultaneously the efficiency, quality, and access, denying the idea that there is a trade-off between them. On the other hand, another group of authors, including Gok and Sezen (2013), Gorgemans et al. (2018), and Varabyova et al. (2017), showed less confidence in the results claiming that in some situations, the trade-off might exist. According to Mitropoulos (2021), that trade-off is clear, and thus improving efficiency should result in a downgrade of quality.

The fact that the literature is unclear regarding this topic, reinforces the need to extend the investigation suggested by this paper. Having a clear notion of the way that quality and access influence the efficiency of healthcare facilities is fundamental to ensure that operations are running optimally, favouring both users and managers. To do so, we considered several variables to evaluate quality and access, and to determine and quantify their impact on the hospital efficiency. Network Data Envelopment Analysis (NDEA) was the method selected to perform the analysis due to its ability to consider the internal structure and connections of complex systems, such as hospitals.

DEA, and other DEA augmented methods, are the most widely used non-parametric models to assess the efficiency of a system (Blatnik et al., 2017; De Nicola et al., 2014; Gautam et al., 2013). In fact, according to Mitropoulos et al. (2013b), “*DEA is more appropriate for the analysis of public sector activities*.” Moreover, Gok and Sezen (2013) and Kao et al. (2021) support that DEA is the most used non-parametric method when estimating efficiency in healthcare services and systems worldwide. Agreeing with the idea of Pai et al. (2019), who stated that DEA is a valuable tool to support management evaluations, the model can suitably evaluate the policies’ impact or detect inefficiencies across all economic fields.

NDEA presents several advantages as a more recent extension of the classical DEA. Following Kawaguchi et al. (2014) and Khushalani and Ozcan (2017), one of the most critical features of NDEA (compared to other DEA-based approaches) is its ability to consider the internal structure of the system. This makes it impossible for a Decision Making Unit (DMU) to be considered efficient if any subdivisions are not (Tone & Tsutsui, 2009). Moreover, a hospital is a complex system whose reality is not easily translated into a mathematical model due to the existence of products between its subdivisions. As stated by Kujawska (2018), NDEA allows taking internal links into consideration, which is fundamental to achieve stronger results.

According to Ferreira et al. (2020), researchers that seek to find the link between efficiency and quality/access in hospital care tend to resort to two main approaches: (1) to consider social dimensions as separated from the production process of outputs using inputs, i.e., efficiency; and (2) to consider that those dimensions should figure out alongside the outputs as the result of inputs utilisation, i.e., as extra outputs. The first alternative does not seem to be appropriate because it assumes that inputs only contribute to produce some outputs (e.g., patients seen in hospital), regardless of the result (e.g., patients live or die within the hospital). In other words, quality and access act as external or non-discretionary dimensions, only explaining the levels of (in)efficiency. Because of that, authors usually use techniques like multiple linear regression analysis, with or without bootstrapping, or even more complex solutions (order-*m* and order-$\alpha$). The second alternative looks more adequate if and only if one is fully aware of the type of impact (positive or negative) that each social dimension might have on performance. However, typically one does not know it. The problem exacerbates when authors use volume-based variables alongside ratios or, even worse, ordinal data (as for the case of patient satisfaction). Considering the drawbacks mentioned above, the proposed NDEA model surpasses those issues and thus can be seen as a third alternative to help the debate regarding the relationship between efficiency and social dimensions.

This paper is structured as follows. Section 2 introduces the state of the art research regarding the type of analysis and methods used by other authors to accomplish the same or similar objectives. Section 3 describes the adopted methodology. Section 4 introduces the case study itself, the proprieties of the Portuguese NHS, the variables used, the models proposed, the data collection and treatment. Section 5 presents the results obtained for all the analyses performed in this paper, followed by their brief description. Finally, Sect. 6 reflects on the results, comparing them with the relevant literature and withdrawing the relevant implications. Also, the limitations and future work are mentioned.

## Literature review

The discussion around the trade-off between efficiency and quality is not a novelty in the healthcare field. Over the last few years, several authors have relied on diverse methods to evaluate whether this link exists.

Benchmarking methods can be either parametric or non-parametric. The parametric approach relies on Stochastic Frontier Analysis (SFA) to calculate hospital efficiency. SFA is an econometric technique that bases the efficiency values on the regression of a stochastic frontier function, bearing in mind the relation between the inputs and outputs of the model. As suggested by Blatnik et al. (2017) and Gautam et al. (2013), this technique presents its advantages, namely the ability to detect the weight of white noise (data errors) on the estimation of efficiency and, consequently, distinguish the noise from hospital inefficiency. However, some drawbacks have also been brought up in the healthcare field when using SFA. Firstly, according to De Nicola et al. (2014), this method requires that the stochastic function is defined a priori, which could highly affect the efficiency estimation and therefore introduce changes in the ranking of hospitals. Secondly, following Gautam et al. (2013), this technique cannot deal with multiple input–output systems, like hospitals, which deviate the models from the reality in which each hospital acts like a single system. These drawbacks and others lead the majority of authors, such as Mitropoulos et al. (2013b), Blatnik et al. (2017), De Nicola et al. (2014) and Kao et al. (2021), to choose a non-parametric approach, mostly DEA, when measuring the efficiency of hospitals and other healthcare facilities. DEA and other related models have the advantage of being *data-driven*, so the frontier is constructed without major assumptions (e.g., defining *a priori* a stochastic function). Additionally, they can easily account for multiple inputs and outputs. More recently, the NDEA can also consider the intricate relationships between different stages within the same system, as with hospitals. A complete comparison between the SFA and DEA, pointing out the advantages and disadvantages in each case, has been done by Jacobs et al. (2006) and Coelli et al. (2005).

A summary of the relevant literature review is presented in Table 7. The inputs, outputs, and other variables selected, as well as the conclusions reached by the authors, differ significantly, creating controversial results regarding the influence that quality and access have on overall efficiency. While some studies, such as the work of Campanella et al. (2017), Chatfield (2014) and van Ineveld et al. (2016), support that efficiency and quality go hand in hand, which entails that if efficiency is improved, quality would improve as well. Other authors, such as Mitropoulos (2021), point out the opposite direction, suggesting that a better efficiency is obtained by decreasing quality. However, the majority of authors did not obtain results clear enough to draw solid conclusions regarding the trade-off. For example, Gok and Sezen (2013) report that in smaller hospitals, there might exist a trade-off between efficiency and the social dimensions; on the other hand, regarding larger hospitals, this trade-off might be questionable. Gorgemans et al. (2018) state that efficiency might evolve in the same way that quality, supporting the nonexistence of the trade-off, however emphasising that this could not be valid for all cases. Kittelsen et al. (2015) defend that there is no clear trade-off. Also, Varabyova et al. (2017) affirm that the existence of trade-offs depends on how quality is incorporated in the models, which could result in a trade-off according with some approaches. On top of that, the majority of the studies in the field are not focused on establishing a relationship between efficiency, quality and access but rather on evaluating the efficiency that healthcare systems operate on. Nevertheless, these studies also rely on several variables to evaluate the hospitals’ efficiency.

The variables chosen to evaluate efficiency are in line with the ones also mentioned in Table 7. As selected by Yang and Zeng (2014) and Almeida et al. (2015), the number of beds, doctors, and nurses remain the most frequent ones, followed by operation costs adopted by Pai et al. (2019) and Mitropoulos et al. (2013b). Regarding the system’s outputs, as appointed by Kao et al. (2021) and Gok and Sezen (2013), the number of inpatients discharges, outpatient visits and surgeries are the most common in the literature.

Regarding the variables used to include quality and access, the most common are the ones related to mortality, readmissions and time spent in the inpatient service, as selected by Chang et al. (2011) and Khushalani and Ozcan (2017). Nevertheless, other authors, such as Gorgemans et al. (2018), also choose the number of infections and procedure complications. Although still present in the literature, a less frequent choice, is the use of the number of hip-fractures surgeries in less than 48 h and the number of outpatient surgeries when it is possible to avoid inpatient admission. It is vital to acknowledge that only countries that follow a Beveridge health model, such as Portugal since 1979, tend to offer to their population a public NHS, and for that reason are interested in guaranteeing equal access to all the population in need. Because these countries are somewhat scarce, the variables used do not evaluate the access dimension in most cases. Indeed, Ferreira and Marques (2019) already commented that there were just a few studies dealing with the link or possible trade-off between efficiency and access in healthcare.

Additionally, Mitropoulos et al. (2013a) refer to the importance of considering the waiting times and the appropriateness of procedures when evaluating healthcare facilities.

All the studies referred to above resorted to a simple DEA model, a two or more stages model or an adjustment technique to incorporate quality indicators in their models. Some also rely on further regression analysis to take into consideration the quality of care. Although these methodologies allow for quantifying the impact of quality on efficiency, they all present some limitations, as referred to in Sect. 1.

The use of NDEA to study this topic is extremely scarce in the literature. Consequently, its application to healthcare could help the discussion around the impact of quality and access on efficiency. Kawaguchi et al. (2014) were the first to apply a Dynamic NDEA in healthcare. However, they only studied the efficiency of Japanese hospitals considering that all quality and access parameters were equal between hospitals. Since that claim might not be valid, the authors recognise the importance of including variables that evaluate quality and access in this type of assessment. The same rationale was applied by Pereira et al. (2021), who relied on NDEA to evaluate the efficiency of Portuguese hospitals, and with that, identify the ones operating on their optimal scale and the ones which do not. However, that evaluation was made disregarding the quality and access dimensions, which could lead to hospitals being classified as efficient while providing low-quality services. Additionally, Kujawska (2018) and Ozcan and Khushalani (2017) also used NDEA and Dynamic NDEA, respectively, in healthcare, but to study the impact of public health on the hospital efficiency. Although the focus of the work is not entirely the same, the variables chosen to evaluate the efficiency of hospitals were in line with the ones reported by other authors, including the number of beds and clinical personnel.

Also, Khushalani and Ozcan (2017) and Mitropoulos (2021) rely on a Dynamic NDEA and a two-stage NDEA model, respectively, to evaluate the relationship between efficiency, quality, and access. The former concludes that there is no trade-off between efficiency and quality, while the latter supports the opposite. Although both authors used a network model, the variables selected in both cases to incorporate quality were based on forms completed by the healthcare services users. This raises a problem since this type of data is highly subjective, as it is based on subjective assessments rather than objective indicators. The inclusion of such data in mathematical models have been strongly criticised by some authors; see Ferreira et al. (2021) for a survey.

## Methodology

The approach presented in this paper follows the network slack-based model proposed by Tone and Tsutsui (2009), modified with the Kuosmanen (2005)’s suggestion to deal with undesirable outputs. This choice was made to tackle two limitations of previous studies.

First, NDEA can be presented in various manners. On the one hand, there can be radial models, and on the other, non-radial models. The radial models assume that the same contraction applied for one input/output/link is applied to the rest due to one fixed variable for all inputs/outputs/links. Contrarily, non-radial models relax this constraint transforming the inequations in equations by adding slacks and eliminating the fixed variable, allowing the inputs/outputs/links to contract independently. In healthcare, there is no guarantee that inputs, outputs, or links change proportionally within them. If that would occur, for example, an increase in beds would mean a proportional increase in doctors. Since it is not necessary to happen, a non-radial model was considered here. According to Tone and Tsutsui (2009), if that proportionally is not guaranteed, only the non-radial approach will provide reliable results. Other authors have used this methodology, including Cheng and Gao (2015) and Kao (2009). However, it is essential to mention that most DEA and NDEA-based studies tend to disregard this issue, thus relying on radial models.

Second, in healthcare, exclusively producing desirable outputs, which aim to be maximised, is not, unfortunately, always possible. Along with the process of treating patients, undesirable outputs should likely appear. These unwanted outcomes should be minimised or even erased from the production process. Due to that, this type of output cannot be treated as desirable ones. It is vital to take into consideration that when using DEA [including the approach presented by Tone and Tsutsui (2009)], the method will try to minimise inputs and maximise outputs. That is why an alteration has been made to the original methodology, which minimises the group of undesirable outputs.

Undesirable outputs in DEA have been dealt with in several different ways that guarantee that the model will minimise these. According to Kuosmanen (2005), among others, the most used methods are to consider them as an input or apply the same mathematical approach as for the inputs to each given set of outputs individually.

Both solutions referred to above present inconsistencies regarding the axioms of production theory, some physical laws, and mathematical flaws, as stated by Färe and Grosskopf (2003) and Kuosmanen (2005). Consequently, to avoid the stated problems, the undesirable output weak disposability suggestion of Kuosmanen (2005), also used in other contexts, was combined with the work of Tone and Tsutsui (2009) and originated the approach used in this paper.

To take into consideration the output weak disposability theory, an abatement factor, $\pmb {\theta }^k$, was added to the model’s original constraints, defined by Tone and Tsutsui (2009). Let a DMU *o* be composed of *K* subunits, each one with a performance score, $\rho ^k_o$; thus, the overall performance score is the average of the *K* subunits; see Eq. (1). As reinforced by Tone and Tsutsui (2009), these efficiencies are unit-invariant, which means that they are independent of the units used to measure the variables in each subunit (inputs, outputs, and links).

1 $$\begin{aligned} \rho _o^*\ =\ \frac{\sum _{k=1}^{K} \rho _o^k}{K},~k=1,\ldots ,K. \end{aligned}$$

Meanwhile, the performance of each subunit of DMU *o* depends on the $m_k$ received external inputs, ${\textbf{x}}^k_o$, the $r_k$ produced outputs going outside of the system, ${\textbf{y}}^k_o$, and the links, ${\textbf{z}}^{(k,h)}_o$. The latter are nothing but the outputs produced in the subunit *k* and flow to the subunit *h*, acting as some of its inputs. Equations (2) and (3) present the original linear programming-based NDEA model proposed by Tone and Tsutsui (2009). The ${\textbf{s}}^{k-}$ and ${\textbf{s}}^{k+}$ represent, respectively, the input and output slack vectors for the subunit *k*.

2 $$\begin{aligned}{} & {} \rho _o^k\ =\ \min _{\lambda ^k,s^{k-}} \quad 1\ -\ \frac{1}{m_k}\sum \nolimits _{i=1}^{m_k} \frac{s_i^{k-}}{x_{io}},~\text {if}~k=1,\nonumber \\{} & {} \rho _o^k\ =\ \max _{\lambda ^k,s^{k+}} \quad \frac{1}{1\ +\ \frac{1}{r_k}\sum \nolimits _{r=1}^{r_k}\frac{s_r^{k+}}{y_{ro}^k}},~\text {if}~k>1, \end{aligned}$$

3 $$\begin{aligned} s.t. \qquad{} & {} {\textbf{x}}_o^k = {\textbf{X}}^k\pmb {\lambda }^k+{\textbf{s}}^{k-},~ k=1,\ldots ,K, \nonumber \\{} & {} {\textbf{y}}_o^k = {\textbf{Y}}^k\pmb {\lambda }^k-{\textbf{s}}^{k+}\,~ k=1,\ldots ,K,\nonumber \\{} & {} {\textbf{Z}}^{(k,h)}\pmb {\lambda }^h={\textbf{Z}}^{(k,h)}\pmb {\lambda }^k,~\forall k,h=1,\ldots ,K,~k\ne h, \nonumber \\{} & {} \sum \pmb {\lambda }^k=1,~ k=1,\ldots ,K,\nonumber \\{} & {} \pmb {\lambda }^k\geqslant {\textbf{0}},\ {\textbf{s}}^{k-}\geqslant {\textbf{0}},\ {\textbf{s}}^{k+}\geqslant {\textbf{0}},~ k=1,\ldots ,K. \end{aligned}$$

On top of that, the outputs of the system were also separated into two different vectors, one representing the desirable outputs, **v**, and another the undesirable outputs, **u**, which resulted in Eq. (4) and (5), after applying the transformation of Kuosmanen (2005) and Kuosmanen and Podinovski (2009).

4 $$\begin{aligned} \begin{aligned}&\rho _o^k\ =\ \min _{\lambda ^k,s^{k-}} \quad 1\ -\ \frac{1}{m_k}\sum \nolimits _{i=1}^{m_k} \frac{s_i^{k-}}{x_{io}},~\text {if}~k=1,\\&\rho _o^k\ =\ \max _{\lambda ^k,s^{k+}} \quad \frac{1}{1\ +\ \frac{1}{r_k}\sum \nolimits _{r=1}^{r_k}\frac{s_r^{k+}}{v_{ro}^k}},~\text {if}~k>1, \end{aligned} \end{aligned}$$

5 $$\begin{aligned} s.t. \qquad{} & {} {\textbf{x}}_o^k = {\textbf{X}}^k\pmb {\lambda }^k+{\textbf{s}}^{k-},~ k=1,\ldots ,K, \nonumber \\{} & {} {\textbf{v}}_o^k = {\textbf{V}}^k\pmb {\lambda }^k\pmb {\theta }^k-{\textbf{s}}^{k+},~ k=1,\ldots ,K, \nonumber \\{} & {} {\textbf{u}}_o^k = {\textbf{U}}^k\pmb {\lambda }^k\pmb {\theta }^k,~ k=1,\ldots ,K, \nonumber \\{} & {} {\textbf{Z}}^{(k,h)}\pmb {\lambda }^h={\textbf{Z}}^{(k,h)}\pmb {\lambda }^k,~\forall k,h=1,\ldots ,K,~k\ne h, \nonumber \\{} & {} \sum \pmb {\lambda }^k=1,~ k=1,\ldots ,K, \nonumber \\{} & {} \pmb {\lambda }^k\geqslant {\textbf{0}},\ {\textbf{0}}\leqslant \pmb {\theta }^k\leqslant {\textbf{1}},\ {\textbf{s}}^{k-}\geqslant {\textbf{0}},\ {\textbf{s}}^{k+}\geqslant {\textbf{0}},~ k=1,\ldots ,K. \end{aligned}$$

However, the problem became non-linear due to the multiplication between the intensity $\pmb {\lambda }$ and the abatement $\pmb {\theta }$ vectors, which made it impossible to solve using linear programming. The linearisation proposed by Kuosmanen (2005) was adopted to overcome that issue, which decomposes the intensity vector into two and redefines the abatement factor as shown by Eq. (6) and (7).

6 $$\begin{aligned} \pmb {\lambda }^k=\pmb {\mu }^k+\pmb {\varphi }^k \end{aligned}$$

7 $$\begin{aligned} \pmb {\theta }^k=\frac{\pmb {\varphi }^k}{\pmb {\varphi }^k+\pmb {\mu }^k} \end{aligned}$$

Therefore, it is possible to linearise and solve the model in (5). In this case, the objective function in Eq. (4) does not change. However, the constraints are as follows:

8 $$\begin{aligned}{} & {} {\textbf{x}}_o^k = {\textbf{X}}^k(\pmb {\mu }^k+\pmb {\varphi }^k)+{\textbf{s}}^{k-}, ~k=1,\ldots ,K, \nonumber \\{} & {} {\textbf{v}}_o^k = {\textbf{V}}^k\pmb {\varphi }^k-{\textbf{s}}^{k+}, ~k=1,\ldots ,K, \nonumber \\{} & {} {\textbf{u}}_o^k = {\textbf{U}}^k\pmb {\varphi }^k, ~k=1,\ldots ,K,\nonumber \\{} & {} {\textbf{Z}}^{(k,h)}(\pmb {\mu }^h+\pmb {\varphi }^h)={\textbf{Z}}^{(k,h)}(\pmb {\mu }^k+\pmb {\varphi }^k),~\forall k,h=1,\ldots ,K,~k\ne h, \nonumber \\{} & {} \sum (\pmb {\mu }^k+\pmb {\varphi }^k)=1, ~k=1,\ldots ,K,\nonumber \\{} & {} \pmb {\mu }^k\geqslant {\textbf{0}},\ \pmb {\varphi }^k\geqslant {\textbf{0}},\ {\textbf{s}}^{k-}\geqslant {\textbf{0}},\ {\textbf{s}}^{k+}\geqslant {\textbf{0}}, ~k=1,\ldots ,K, \end{aligned}$$

9 $$\begin{aligned} \text {where} \qquad \qquad{} & {} {\textbf{X}}^k = ({\textbf{x}}_1^k,\ldots ,{\textbf{x}}_n^k) \ \in {\mathbb {R}}^{n \times m_k} \nonumber \\{} & {} {\textbf{V}}^k = ({\textbf{v}}_1^k,\ldots ,{\textbf{v}}_n^k) \ \in {\mathbb {R}}^{n \times r_k} \nonumber \\{} & {} {\textbf{U}}^k = ({\textbf{u}}_1^k,\ldots ,{\textbf{u}}_n^k) \ \in {\mathbb {R}}^{n \times w_k} \nonumber \\{} & {} {\textbf{Z}}^{(k,h)} = ({\textbf{z}}_1^{(k,h)},\ldots ,{\textbf{z}}_n^{(k,h)}) \ \in {\mathbb {R}}^{n \times t_{(k,h)}} \nonumber \\{} & {} \pmb {\mu }^k = (\mu _1^k,\ldots ,\mu _n^k) \ \in {\mathbb {R}}^{n}_+ \nonumber \\{} & {} \pmb {\varphi }^k = (\varphi _1^k,\ldots ,\varphi _n^k) \ \in {\mathbb {R}}^{n}_+ \end{aligned}$$

Bear in mind that the problem is presented under Variable Return to Scale (VRS) but could easily be converted into a Constant Return to Scale (CRS) problem by removing the last constrain from Eq. (8).

## Case study: the Portuguese NHS

The proposed methodology was applied to the Portuguese public hospitals belonging to the NHS. Nine efficiency, seven quality, and three access-related variables were chosen to perform the analysis, using four original models and yearly data retrieved from 2016 to 2019. More recent data was disregarded because of the COVID-19 pandemic outbreak, which forced the adoption of unusual practices in hospitals worldwide. Further research in this topic is necessary, though.

### An overview

Healthcare in Portugal includes several types of providers, being the Portuguese NHS the largest one. Nevertheless, a smaller percentage of the population relies on private providers, often associated with private voluntary insurance schemes or other subsystems specific to certain professions (Ferreira et al., 2013, 2018).

The Portuguese NHS was founded in 1979 (Law number 56/79, 15th September) following a Beveridge model. To avoid an abusive use of the system and contribute to its funding, a co-payment is required in some services for some patients according to their social and financial situation. Since most funds come from the Central Government (after taxation), the Portuguese NHS, which includes all public primary and secondary healthcare entities, is characterised as a not-for-profit system, universal, general, and equitable for all Portuguese citizens regardless of their capability to pay for the treatments (Ferreira et al., 2013; Ferreira & Marques, 2019). Therefore, although these public services should have been mostly concerned with the social dimensions of care, the truth is that financial sustainability (efficiency) should also be considered a serious concern by healthcare providers.

The creation of the NHS followed the end of the dictatorship regime that prevailed in Portugal until 1974 with the goal of transferring to the government the duty of providing, financing, managing, and regulating healthcare services. After its creation, several modifications have been introduced to satisfy the patient expectations and keep up with the fast development of medicine over the last years. Besides, the expenditure on healthcare started to rise around the end of the last century threatening the NHS sustainability and its capability to continue providing equitable access.

Nowadays, in Portugal mainland there are 22 hospital centres (groups of secondary care hospitals horizontally integrated), and eight local health units (vertical aggregations between primary care facilities and secondary care hospitals), while 13 hospitals remain single entities. All of these are public hospitals. There is currently (February 2022) one hospital providing services to the NHS throughout a public-private partnership. Due to their autonomy, the archipelagos of Azores and Madeira have different healthcare services that do not follow completely the guidelines of the Ministry of Health and were consequently excluded from this study. Local health units and specialised hospitals (oncology centres) were also disregarded from the analysis because of their unique production process, turning them incomparable to the others.

As the expenditure with healthcare in Portugal continues to raise, the importance of keeping the system as efficient as possible also raises. Portugal is one of the countries that spends more of its Gross Domestic Product (GDP) per year with healthcare, roughly 10% of the national GDP. This significant percentage of public money increases the pressure on the government to keep the costs as low as possible without jeopardising the quality and availability of care provided. To contain the rise observed over the last years, the government should find new ways to improve efficiency and achieve better outcomes with the same or fewer resources (Pereira et al., 2020).

### Efficiency

The most frequent inputs used in DEA to evaluate efficiency are related to capital investment, labour, and expenditures (Kao et al., 2021; van Ineveld et al., 2016; Yaya et al., 2020). Particularly, the use of the number of beds is widely chosen to evaluate capital investment (Chang et al., 2011; Gao & Wang, 2020; Gok & Sezen, 2013), the number of doctors and nurses (or their financial impact) to evaluate labour (De Nicola et al., 2014; Gorgemans et al., 2018; Varabyova et al., 2017), and the operating costs to account for expenses (Chatfield, 2014; Ferreira et al., 2020; Khushalani & Ozcan, 2017). Regarding the outputs, as seen in Sect. 2, the number of patients treated (inpatient and outpatients) is the most used measure of production (Gao & Wang, 2020; Gorgemans et al., 2018; van Ineveld et al., 2016). Nevertheless, others have frequently been used, such as the number of surgeries (Chang et al., 2011; Gok & Sezen, 2013; Ferreira et al., 2020), when the analysis considers these services.

To ensure a more detailed set of results, the efficiency variables were separated into two groups. The differences are the substitution of the number of doctors and nurses by its financial impact, the personnel costs, and the addition of the outsourcing costs in some models. Note that only one group was considered at each time. For example, when the model considers the number of doctors and the number of nurses, the costs with personnel were not applied.

The selected variables to consider efficiency measures in the models are presented in Table 1, along with their description.

**Table 1:** Selected variables to take into consideration the efficiency of healthcare and their description

Variable	Description
Number of beds (E1)	Number of beds available in the inpatient services (wards) of each hospital, excluding nursery, individual rooms, continuous care, palliative care and psychiatry beds
Number of doctors (E2)	The number of regular yearly hours of full-time equivalent doctors working in each hospital (including overtime)
Number of nurses (E3)	The number of regular yearly hours of full-time equivalent nurses working in each hospital (including overtime)
Operational costs (E4)	The yearly non-capital expenses (excluding costs with staff) reported by each hospital to keep the production line working, in this case, to keep treating patients
Costs with personnel (E5)	The yearly expenses with hospital staff salaries (including subsidies)
Costs with outsourcing (E6)	The part of each hospital yearly expenses that concerns the cost of outsourcing services, such as utilities, security, representation expenses and additional seasonal staff wages
Inpatients treated (E7)	The number of inpatients discharged from the hospital, excluding service transfers within the same hospital, nursery, individual rooms, continuous care, palliative care, and psychiatry discharges
Outpatients treated (E8)	The number of outpatients appointments realised, including emergency appointments
Number of surgeries (E9)	The total of non-urgent major (requiring overnight) and nonurgent ambulatory (not requiring overnight) surgeries

### Quality

To evaluate quality in healthcare, both the safety dimension and the appropriateness of the care provided are fundamental (Gorgemans et al., 2018; Ferreira & Marques, 2019; Mitropoulos, 2021). Consequently, the variables found in the literature related to the appropriateness of the care (Q1–Q3) (Ferreira & Marques, 2019; Khushalani & Ozcan, 2017; Onder et al., 2021) and procedures safety (Q4–Q7) (Ferreira et al., 2018; Gorgemans et al., 2018; van Ineveld et al., 2016) were added to the analysis. The quality variables presented in Table 2 aggregate both the desirable (Q1–Q3) and the undesirable ones (Q4–Q7).

### Access

Table 3 presents the list of the selected variables to evaluate the access to healthcare as well as their description. This selection, which simultaneously shows the desirable variable (A1) and the undesirable ones (A2 and A3), results from the fusion of the variables found in the literature used to evaluate the availability of the services, an essential dimension of the care provided according to Mitropoulos et al. (2013a).

**Table 2:** Selected variables to take into consideration the quality of care and their description

Variable	Description
Patients not readmitted within 30 days after discharge (Q1)	The number of inpatients discharged that do not return to the hospital for an unexpected readmission within 30 days after leaving
Inpatient time under 30 days (Q2)	The number of inpatients that do not stay in the inpatient’s service more than 30 days between the check-in and discharge
Outpatient surgeries on potential outpatient procedures (Q3)	When a surgery is classified as minor (ambulatory), it can be done in the outpatient service or in the operating theatre. The latter case may raise the risk of nosocomial infections, thus the former is always preferable
Number of decubitus/pressure ulcers (Q4)	The number of decubitus ulcers occurrences in the inpatient service
Number of bloodstream infections related to central venous catheter (CVC) (Q5)	The number of bloodstream infections associated with an infection resulting from the existence of a CVC
Postoperative sepsis/septicaemia cases (Q6)	The number of occurrences of sepsis after major or minor surgery
Postoperative PE or DVT cases (Q7)	The number of occurrences of a Pulmonary Embolism (PE) of Deep Vein Thrombosis (DVT) after major or minor surgery

**Table 3:** Selected variables to take into consideration the access to healthcare and their description

Variable	Description
Hip fracture surgery in less than 48 h (A1)	The number of patients older than 65 diagnosed with a hip fracture and forwarded to surgery in less than 48 h
First appointment not in legal expected time (A2)	The number of outpatients visits that occurs after the expected time for the first appointment. In the Portuguese NHS, each group of diagnosis have a maximum legal time of response for a first appointment in hospitals. This variable accounts for the number of patients who did not have their appointment within the legal time period, and so, represents the number of patients who have seen their access to care delayed or postponed
Surgery waiting time (A3)	The number of days before major surgery in each hospital

### Models

**Fig. 1:**
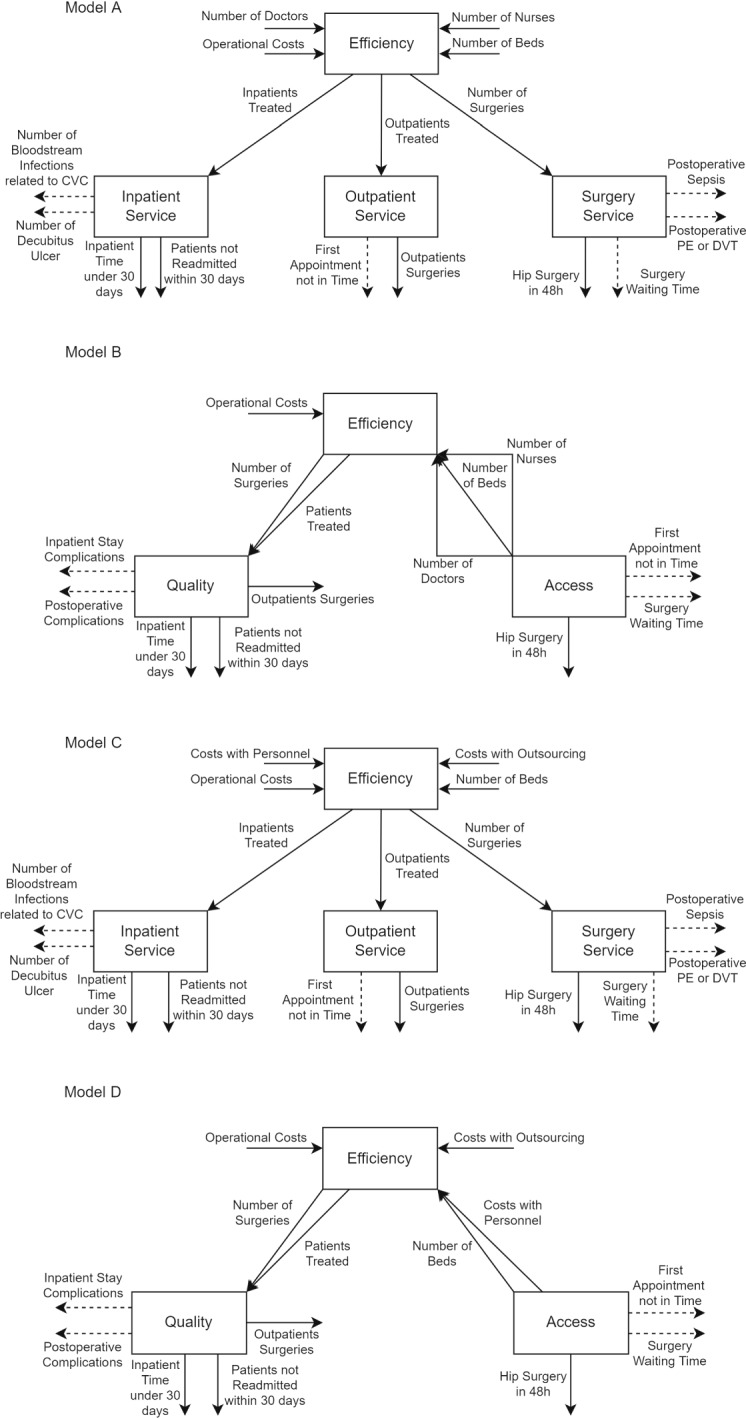
Models representation. Each rectangle represents a subunit and each arrow an input (pointing to a subunit), output (pointing out from a subunit) or link (between subunits). The outputs can be desirable, represented by the solid lines, or undesirable, represented by the dashed lines

The variables referred to above were used to create four NDEA models (A, B, C, and D), as shown in Fig. 1. The models suggested have a different structure, and the variables considered in each also change.

In the models presented, each rectangle represents a subunit, and the group of all subunits represents a hospital. The arrows pointing from outside to a subunit represent an input variable, the arrows between subunits a link, and the arrows pointing out from a subunit an output. The outputs can be desirable, represented by the solid lines, or undesirable, represented by the dashed lines.

Model A divides each DMU into four subunits, one representing the efficiency of the hospital (the administrative part of operations, $k=1$) and the others representing different services provided by that hospital ($k=2,3,4$). On the other hand, model B divides each DMU into three subunits, each one representing the dimensions that this study intends to evaluate, efficiency ($k=1$), quality ($k=2$) and access ($k=3$).

To incorporate the financial variables selected, *Costs with Personnel (E5)* and *Costs with Outsourcing (E6)*, another two models were also evaluated. Model C, which is the same as model A but with variable E5 instead of E2 and E3, and the addition of E6 to the efficiency subunit; and model D that results from of the same alterations to model B.

### Data

The data used to calculate the overall efficiency score and the divisional scores of each DMU was collected from the Ministry of Health of the Government of the Portuguese Republic. In particular, from the benchmarking database of the *ACSS—Administração Central do Sistema de Saúde, IP*^1^ and the database *Transparência — Sistema Nacional de Saúde*.^2^ The data collected concerns the period between 2016–2019 (4 years).

The *Administração Central do Sistema de Saúde* provides the data organised by groups, B to F, according to the characteristics and similarities between the healthcare entities (hospitals, hospital centres and local health units). Furthermore, hospitals that belong to the same group are expected to receive the same complexity of patient cases and perform the same level of treatments, consequently, a rigorous analysis implies that comparisons should only be done within the same group.

From the 45 healthcare entities that made up the (mainland) Portuguese NHS, five were eliminated due to its particularities regarding the healthcare provided (three oncology centres and two psychiatric hospitals); eight local health units, since they include primary care facilities, were also not considered; two hospitals operating under a public-private partnership, as well as, other two that followed the same management method in the time period considered, were also discarded and so was another hospital, due to incomplete data sets. Brief statistic information regarding the data used is presented as attachment.

When performing DEA calculations, the relation between the number of variables and the number of DMUs could impact the results, particularly the number of DMUs that are considered efficient. To obtain a more detailed set of results (fewer DMUs with a score of 1), the number of DMUs should be as significant as possible, and the number of variables reduced.

Furthermore, variables that present a significant correlation between them should not be applied simultaneously in the same model, in order to improve the results. A Pearson’s Correlation test was performed to evaluate the possible correlation between variables.

The test analyses and quantifies the level of correlation between two variables. If a pair of variables present a correlation of over 95%, then their impact in the model is very similar, and due to that, one of them can be removed without jeopardising the results. It is important to acknowledge that only pairs of variables with the same classification (input, output or link) within the model can be reduced to one.

## Results

The results were obtained using the MATLAB^®^ computing platform (version 2018b) with the addition of the Optimisation Toolbox. The models suggested were implemented into the platform and applied to the selected data. The relevant results are presented in the sections below, as well as a sensitivity analysis.

### Pearson’s correlation test

The Pearson’s correlation test results were computed by pairing all the variables used in each model and by calculating the Pearson’s correlation score.

As mentioned previously, only pairs of variables that influence the same subunit and have the same classification (input, output or link) within that model present a significant result.

This test identified three clusters of variables that have a correlation score higher than 95%. The first one aggregates the variables *Patients not Readmitted within 30 days (Q1)* and *Inpatient Time under 30 days (Q2)*; the second groups the *Operational Costs (E4)* with *Costs with Personnel (E5)* and *Costs with Outsourcing (E6)*; and the third clusters the *Number of Doctors (E2)*, *Number of Nurses (E3)* and *Operational Costs (E4)*. Regarding the last set of variables, to avoid loss of information while evaluating the models, an approach used by Ozcan and Khushalani (2017) and Yaya et al. (2020) was adopted. This consisted of combining the *Number of Doctors (E2)* and the *Number of Nurses (E3)* into one single variable, the *Clinical Staff*. Although the work of doctors and nurses are not comparable, both variables measure the number of hours spent by part of the workforce in a healthcare facility. As stated by Kujawska (2018), Ozcan and Khushalani (2017) and van Ineveld et al. (2016), labour is essential to be accounted for when estimating a hospital efficiency; however, that measure does not need to be divided into working classes to produce reliable results.

### Real data results

The hospitals’ efficiencies were calculated using the four different models proposed and the data retrieved from the four years being analysed. Since several variables were classified as highly correlated, various estimations were performed for each model, one for each different possible combination of variables.

On top of the VRS efficiency, also the CRS and scale efficiency were calculated for all DMUs. The complete set of results can be found in Appendix 1. Nevertheless, the aim of this study is to evaluate whether there is or not an impact caused by the quality and access variables on the hospital’s efficiency. For that, external influences on the hospital’s operation should be reduced to guarantee that each DMU’s efficiency is calculated assuming that the hospital operates on its optimal scale. Since the Portuguese hospitals operate under a centralised budget constraint, according to Mitropoulos et al. (2013b), considering the CRS efficiency might induce in error. Furthermore, Alonso et al. (2015) also state that since the market competition between hospitals is imperfect in an NHS, and the regulatory measures significant, VRS should be the efficiency score considered to ensure a more accurate analysis.

**Fig. 2:**
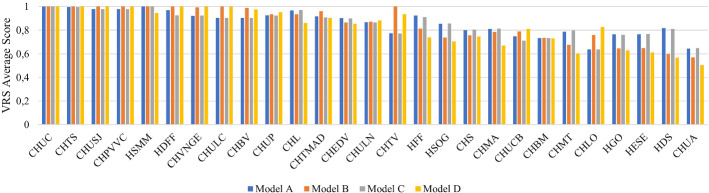
VRS efficiency score average for each DMU between 2016 and 2019

Accordingly, the VRS efficiency score average for the period 2016–2019 for each model and DMU is presented in Fig. 2. The model’s variation examined in this set of results considered the variable *Patients not Readmitted within 30 days (Q1)* and for models A and C also the *Clinical Staff* and *Costs with Personnel (E5)*, respectively.

Models A and C report a high similarity between the results, both with two DMUs on the frontier and another three in close proximity to it. The same relationship is verified for models B and D, although the similarity between the efficiency scores is not as pronounced as for models A and C. Nevertheless, both present seven DMUs on the frontier, with another one close to it. The higher number of efficient DMUs for models B and D was already expected due to the number of subunits in each model (four for models A and C, three for models B and D).

Observing the results from a broader perspective, reveals their similarity between the four models, excluding some DMUs such as *Hospital Distrital de Santarém, EPE (HDS)*, *Centro Hospitalar Tondela-Viseu, EPE (CHTV)* and *Hospital da Senhora da Oliveira, Guimarães, EPE (HSOG)*. This finding might imply that estimating the hospitals’ efficiency score with any of the models proposed would lead to equivalent conclusions regarding the impact that each variable related to quality and access provoke in overall efficiency.

### Sensitivity analysis

In addition to the relatedness of the results observed between models, also within the same model, the results obtained for each variation report high similarity rates, in some cases of nearly 100%.

To perform this sensitivity analysis, the VRS efficiencies were calculated several times for the same model, substituting one element of each correlation cluster and therefore creating several combinations between them. This originated four variations of models A and C and two variations of models B and D. The difference in number of variations is a consequence of each variable’s role within the models (input, output or link) since only variables inside the same category can be changed.

For the vast majority of the DMUs, the results obtained^3^ with any variations of model A do not differ significantly. Only two out of 27 DMUs, *Hospital Distrital da Figueira da Foz, EPE (HDFF)* and *Hospital da Senhora da Oliveira, Guimarães, EPE (HSOG)* were detected to present a significant alteration in the efficiency, around 8% and 12%, respectively. Model C also produced a similar sensitivity analysis report, identifying the majority of the DMUs as inducing insignificant changes in the efficiency scores (all below 5%), with the exception of *Hospital Distrital da Figueira da Foz, EPE (HDFF)*, which still presents a significant difference, around 10%, in the results. However, in model C, the first two variations present a worse result when compared with the others, which is the opposite case of model A, where the first two variations present a better result. Regarding models B and D, their sensitivity analysis was close to perfection, with both models only reporting differences below 1% for five DMUs, and no difference for the remaining.

### Hypothetical scenarios

To study the impact that each quality and access variable provoke in the overall hospital efficiency, two scenarios were built.

The first one assumes that every variable is in its best situation, and the second in its worst. The creation of these scenarios involved the alteration of the data used to calculate the efficiency scores of each variable individually, both for its best and worst case. For example, when evaluating the impact of the *Number of Decubitus Ulcers (Q4)*, in the first scenario, all the data (regarding this variable) were transformed into 0’s (close to 0, due to the instability that 0’s can provoke in DEA), since the best scenario regarding decubitus ulcers happens when none of the inpatients has one. The same rationale applies to the worst scenario, which uses the total number of inpatients since it is the maximum number of decubitus ulcers that could happen in each hospital.

**Fig. 3:**
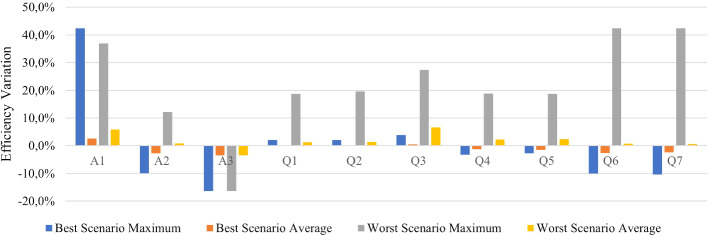
Maximum and average ratio between the hypothetical data and the real data for the period 2016–2019. Results obtained from model A

This process was repeated for each of the ten variables related to quality and access, using the VRS efficiency calculated with model A. The results originate new efficiency scores for each hospital, which, when compared to the real ones (presented in Sect. 5.2), allows to evaluate the impact that an alteration in these variables would have in the overall efficiency. To quantify that impact, the ratio between the hypothetical data and the actual data was calculated. The results are shown in Fig. 3.

Note that if the ratio is equal to one, the change in the variable’s data does not impact hospital efficiency. If the ratio is greater than one, then the new efficiency score is higher than the baseline (actual data), which means that the change of variable results in a better efficiency. On the other hand, if the ratio is smaller than one, then the baseline efficiency is higher than the one calculated with the changed data, so that specific alteration in the variable decreases the overall efficiency.

The information presented in Fig. 3 is the impact that each variable causes. In other words, the excess over one or the deficit to one. This means that the variables with positive results have seen their efficiency increase with the hypothetical data, and those with negative results have seen their efficiency decrease.

For each variable, the graphic shows the maximum and average (for all DMUs) impact that each scenario causes on the hospital’s overall efficiency. Only variable A3 shows a negative result for both scenarios, which means that this variable might already be at its optimal level; however, it impacts the overall efficiency since the average impact (around 4%) is still significant in both scenarios.

On the contrary, variables Q1 and Q2 present a non-significant impact, almost null in the best scenario (2% maximum, 0.1% average), on overall efficiency. Nevertheless, both variables still present a maximum impact of 20% in the worst scenario, which is due to an isolated impact that these variables have in specific DMUs.

Variable Q3 follows the same behaviour for the best scenario, presenting a non-significant average impact of 0.5% and a maximum of 4%. However, this variable in the worst scenario originates an average impact of almost 7%, which is significant, and might imply that the actual data is closer to the best scenario, not producing differences but distanced from the worst scenario.

Regarding the variables, *Number of Decubitus Ulcers (Q4)* and *Number of Bloodstream Infections related to CVC (Q5)* both present a negative impact in the best scenario, which means that the hospital’s efficiency is reduced in this case; and a positive result in the worst scenario, which is the consequence of the increase in the hospital’s efficiency. Moreover, the impact is more noteworthy in the worst case (average around 2.5%) than in the best scenario (average around $-$ 1.5%).

Opposing variables Q6, Q7 and A2, also present a negative impact for the best scenario and a positive one for the worst. However, in this case, the impact is more significant in the first scenario, presenting an average of almost − 3% in the best scenario and an average below 1% in the worst.

Finally, variable A1 is the only one that shows a positive and significant change in the efficiency for both scenarios. Although this change is more remarkable for the worst scenario (average of 6%), it is still relevant in the best scenario (average of 3%).

**Fig. 4:**
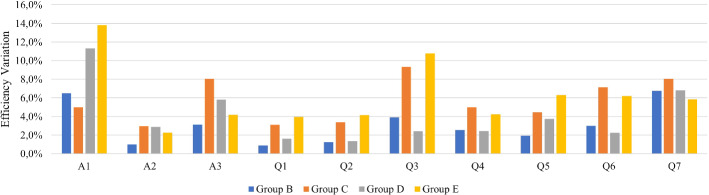
Average absolute variation of efficiency score for each variable and hospital group

The results presented in Fig. 3 do not take into consideration that in Portugal, hospitals are divided into five groups according to their operation plan, size and type of patient treated. This division allows studying the impact that quality and access have in each group. The results of this analysis are presented in Fig. 4.

Figure 4 shows the absolute average impact, of both scenarios, that each variable provokes in each group. For half of the sample, group E, which represents the biggest and most developed hospitals, shows the most significant impact. Following the same behaviour, group B, which represents the smallest and less developed hospitals, shows the least impact for half of the sample.

Nevertheless, the results are not significantly conclusive, not allowing to establish any connection between the size and type of hospital with the impact that quality and access have on their efficiency. As observed, the results are considerably scattered across all variables and groups.

## Discussion and conclusions

The aim of this paper was to evaluate the impact that quality and access have on hospital performance. To achieve it, nine variables related to efficiency, seven related to quality and three related to access were selected to calculate and observe the variation in the efficiency scores of each hospital using a modified NDEA methodology. The data used are quantitative and longitudinal, collected for each year individually, and applied to the same sample of hospitals. All the values retrieved from the database are positive, no missing data was detected, and the values were not standardised. Therefore, the magnitude of the variables differs significantly according to their context.

Nevertheless, the variation of values across the interval considered was reduced, with most variables presenting a change under 10%, except for those related to the inpatient service quality and surgery departments.

It is essential to highlight that all variables used correspond to absolute values to ensure the desired behaviour of the NDEA algorithm. The percentages and ratios retrieved from the database were previously transformed to ensure that no mathematical constraint could impact the results.

Detailed yearly statistic analysis for each variable used can be found in Appendix 1

Take into consideration that the conclusions reached were obtained using data from the Portuguese NHS. As explained before, these conclusions might not be valid for other NHSs, especially those that do not follow the same model as the Portuguese one. Healthcare services based on the Bismack model differ significantly from the Portuguese, which follows a Beveridge model. Therefore, caution is recommended when extrapolating the conclusions to other case studies.

Firstly, a correlation analysis was performed between all the variables to detect possible interactions, which were used to improve the quality of the results. Some of those connections were expected, such as the relationship between the number of doctors, nurses and operational expenses. In general, hospitals hire doctors and nurses proportionally since both vary with the number of beds available in each facility. Moreover, wages are one of the most relevant expenses that all companies must assume, so the impact that the number of personnel produced in the operational costs was not unforeseen.

The analysis identified other two clusters, one that relates the different types of costs, which are associated *a priori* since all relate to financial data regarding each hospital, and another that assembles the quality variables concerning the inpatient service. This last cluster which relates readmissions with time spent in the inpatient service might suggest that the same patients that are admitted unexpectedly more than once in less than 30 days are the ones that stay more extended periods hospitalised, which could correspond to the more complex cases, requiring in general more medical assistance.

To be able to compare the impact of an alteration in a specific variable to efficiency scores, a common baseline of comparison must be defined. Data from the Portuguese NHS between 2016 and 2019 was used to calculate these baseline efficiency scores, employing the four suggested models. The baseline scores correspond to the direct application of the models with the data retrieved from the case study without performing any change.

The baseline efficiency scores obtained regarding the VRS analysis are in line with previous works, such as the findings of Pereira et al. (2021), who evaluated the efficiency of the Portuguese NHS hospitals and detected that out of the 27 DMUs considered, nine were on the frontier. Although the authors obtained a more substantial number of efficient DMUs, the ones that this study considers on the frontier are also on that list. Moreover, the results showed in Sect. 5.2 also support the apparent conclusion regarding the location of these DMUs as suggested by the authors, who mentioned that the majority of efficient DMUs are located near the coastline, especially close to the north and centre urban centres.

Respecting the group division that the hospitals are subjected to by the stakeholders, the results show that smaller hospitals, the ones in group B, and the largest and more advanced ones, group E, are the ones which present more DMUs on the frontier and better scores on the overall efficiency analysis. Nevertheless, all groups have at least one DMU considered efficient, although group D not having any DMU simultaneously considered efficient by the four models.

Apart from the VRS efficiency score, also the scale efficiency score was calculated for each hospital. Instead of considering a hospital as an isolated environment, this measure also considers the impact that the surroundings and characteristics of each hospital have on the efficiency. When comparing the VRS and scale scores, the most relevant difference concerns the scores of group E, which decreased significantly when scale efficiency was calculated.

Group E are the hospitals considered the top in healthcare treatment in Portugal since it is where the most complex and advanced treatments are delivered. Consequently, all the patients with complex cases are diverted from their original hospitals to one of these. As a result, the scale efficiency shows better results for group B, where only simpler procedures are performed and penalise the other hospitals due to the type of care that these facilities provide.

Adding to the comparison with the results of Pereira et al. (2021), the sensitivity analysis performed within each model also reinforces the proposed models’ validity, and the methodology suggested. The analysis was performed to evaluate whether a change in the variables selected, which would affect the observations used in the DEA, would significantly impact the results obtained. Therefore, the same model was used with different combinations of variables, particularly in the efficiency subdivision. The results of the various variations of each model were almost equal, which supports the idea that if other variables were chosen to perform this evaluation, the final conclusions would be similar. Note that even presenting more differences, the comparison of results between different models does not produce contradictory conclusions.

Resorting to the same models, methodology and baseline data, two scenarios were built to evaluate if there is or not an impact of quality and access variables on the hospitals’ overall efficiency. The first scenario assumed that the quality and access variables were in their best performance, while the second assumed their worst situation. This evaluation was performed variable by variable, scenario by scenario to be able to draw conclusions individually regarding each variable.

Only two variables out of the ten considered show a reduced impact on the overall efficiency in both scenarios. The remaining showed, at least in one scenario, a significant change in the efficiency scores, with half of the sample showing a deterioration in efficiency in the best case scenario and an improvement in efficiency in the worst case. In other words, almost the majority of variables suggest that when quality and access are at their best levels, efficiency will be penalised; and when those variables are in their worst situation, efficiency will be improved.

This apparent trade-off between efficiency, quality and access contradicts several other studies, such as the findings of Cheng and Gao (2015) and Khushalani and Ozcan (2017) supporting that this trade-off is nonexistent. In fact, both authors defend that improving quality will directly improve efficiency and that a hospital could strive for a better quality service without jeopardising its efficiency. Also, Campanella et al. (2017) concluded that hospitals could be more efficient without affecting the quality of care already delivered. In Portugal, Ferreira et al. (2020) also agree that there is no trade-off between efficiency and the social variables (quality and access).

Contrastingly, other authors seem to support the results obtained, supporting the idea of a trade-off. Yang and Zeng (2014) are clear when stating that to achieve better quality, a hospital will need to increase its expenses and consequently decrease its efficiency. The findings of Gok and Sezen (2013) are not so clear, supporting that for some hospitals, depending on their size, this trade-off might exist, while for the remaining, it is nonexistent. This uncertainty regarding the existence of a trade-off is also supported by Almeida et al. (2015), who using data from the Portuguese NHS concluded that their findings were insufficient to establish a clear relationship between efficiency and quality. This is owed to part of their results seeming to support it, while another seeming to contradict it. However, also in Portugal, Ferreira and Marques (2019) support the trade-off defending that improving efficiency may result in a decrease in the quality of care, mainly regarding the safety dimension inherent to it. Nevertheless, concerning access to the healthcare system, the authors sustain that there is no trade-off with efficiency.

Regarding the evaluation made, considering the five groups in which the Portuguese hospitals are divided, the results do not show any significant correlation. Contrasting with Gok and Sezen (2013) and Yang and Zeng (2014), who conclude that the trade-off is present in the smallest hospitals but not in the largest ones, the results presented in Fig. 4 seem to point in the opposite direction. Even though the results are not conclusive, they seem to support that the impact on the performance is more pronounced in larger hospitals than in smaller.

Considering all the findings, the results obtained might suggest that quality and access influence the hospital’s efficiency, more specifically in a negative way, implying that there might be a trade-off between them. Nevertheless, the results are not constant across all the variables, which might imply that some quality and access dimensions do not affect the efficiency.

On top of that, this study evaluated each variable individually, which means that only data regarding each variable being study at the given time was changed. In reality, all the variables considered are interconnected, so if one is affected, others will be affected as well. Consequently, to study the real impact that a variable provokes in the overall performance, the alterations introduced with the two hypothetical scenarios should be more robust to closer mimic the reality. With that limitation being highlighted, the apparent relationship that was established between efficiency, quality and access, might not be translated to reality as clearly as the mathematical findings suggest.

Nevertheless, that limitation does not overthrow the possibility of the existence of the trade-off. Thereby, the government should take this into consideration when planning and creating policies that directly impact the national NHS, fomenting measures that guarantee that the quality and access desirable levels are achieved by all the hospitals, even if their efficiency is penalised.

In the future, other methodologies should be used to try to clarify this topic, which is far from being well defined. Moreover, a new approach might be suggested to create scenarios closer to reality and consequently produce more authentic results.

## Appendix A

See Tables 4, 5, 6, 7.

**Table 4:** List of the considered hospitals (DMUs) and their abbreviation used in the results presentation

#	Group	DMU	Abreviation
1	Group B	Centro Hospitalar do Médio Ave, EPE	CHMA
2	Group B	Centro Hospitalar Póvoa de Varzim/Vila do Conde, EPE	CHPVVC
3	Group B	Hospital Distrital da Figueira da Foz, EPE	HDFF
4	Group B	Hospital Santa Maria Maior, EPE	HSMM
5	Group C	Centro Hospitalar Barreiro/Montijo, EPE	CHBM
6	Group C	Centro Hospitalar de Leiria, EPE	CHL
7	Group C	Centro Hospitalar de Setúbal, EPE	CHS
8	Group C	Centro Hospitalar do Baixo Vouga, EPE	CHBV
9	Group C	Centro Hospitalar Entre Douro e Vouga, EPE	CHEDV
10	Group C	Centro Hospitalar Médio Tejo, EPE	CHMT
11	Group C	Centro Hospitalar T$\hat{a}$mega e Sousa, EPE	CHTS
12	Group C	Centro Hospitalar Universitário Cova da Beira, EPE	CHUCB
13	Group C	Hospital da Senhora da Oliveira, Guimarães, EPE	HSOG
14	Group C	Hospital Distrital de Santarém, EPE	HDS
15	Group D	Centro Hospitalar Tondela-Viseu, EPE	CHTV
16	Group D	Centro Hospitalar Trás-os-Montes e Alto Douro, EPE	CHTMAD
17	Group D	Centro Hospitalar Universitário do Algarve, EPE	CHUA
18	Group D	Centro Hospitalar Vila Nova de Gaia/Espinho, EPE	CHVNGE
19	Group D	Hospital Espírito Santo de Évora, EPE	HESE
20	Group D	Hospital Fernando Fonseca, EPE	HFF
21	Group D	Hospital Garcia de Orta, EPE	HGO
22	Group E	Centro Hospitalar de Lisboa Ocidental, EPE	CHLO
23	Group E	Centro Hospitalar e Universitário de Coimbra, EPE	CHUC
24	Group E	Centro Hospitalar Universitário de Lisboa Central, EPE	CHULC
25	Group E	Centro Hospitalar Universitário de São João, EPE	CHUSJ
26	Group E	Centro Hospitalar Universitário do Porto, EPE	CHUP
27	Group E	Centro Hospitalar Universitário Lisboa Norte, EPE	CHULN

**Table 5:** Variables statistics

Variable	Year	Mean	SD	CV (%)	Min	Max	Median
E1	2016	584.6	386.9	66.2	117	1809	445
	2017	591.0	385.7	65.3	117	1807	459
	2018	588.6	377.1	64.1	117	1762	500
	2019	580.3	360.6	62.1	117	1711	503
E2	2016	250,132.1	201,985.6	80.8	31,957	716,248	164,493
	2017	258,294.8	203,489.4	78.8	32,327	721,963	164,343
	2018	269,054.2	208,914.3	77.6	34,009	728,806	177,963
	2019	272,894.3	208,256.5	76.3	41,119	727,317	190,886
E3	2016	426,606.0	301,578.7	70.7	82,153	1,220,376	322,356
	2017	429,526.9	301,262.2	70.1	79,565	1,189,334	328,507
	2018	425,388.2	299,867.3	70.5	78,978	1,201,073	322,968
	2019	425,106.4	300,613.2	70.7	79,682	1,200,446	321,480
E4	2016	155,165,335.7	123,785,037.0	79.8	23,820,404	452,018,313	96,882,770
	2017	162,016,835.5	128,896,298.0	79.6	23,884,991	467,724,538	99,851,907
	2018	174,054,077.8	138,089,107.1	79.3	25,730,553	500,978,922	110,649,358
	2019	185,888,554.0	144,660,138.4	77.8	28,560,022	522,912,625	124,991,054
E5	2016	78,525,848.6	57,745,775.6	73.5	13,291,520	224,613,038	53,463,904
	2017	82,749,683.9	60,115,746.6	72.6	13,705,107	236,698,480	56,623,000
	2018	87,919,787.5	63,718,700.2	72.5	14,968,308	249,881,517	59,410,572
	2019	96,355,372.1	68,133,292.6	70.7	16,923,922	266,927,122	67,818,630
E6	2016	22,371,598.9	14,732,646.4	65.9	3,903,000	61,091,612	17,713,714
	2017	23,121,423.0	15,156,148.6	65.6	4,198,201	63,842,699	17,923,352
	2018	25,169,611.9	16,653,460.1	66.2	4,300,879	70,629,430	20,890,397
	2019	26,970,613.3	17,378,244.2	64.4	4,679,207	67,752,715	23,090,992
E7	2016	22,505.1	12,951.7	57.5	5178	60,205	21,409
	2017	22,018.3	12,712.2	57.7	4923	58,814	20,718
	2018	21,588.7	12,332.9	57.1	5285	58,126	20,674
	2019	21,644.4	12,342.1	57.0	5561	56,514	20,591
E8	2016	330,307.0	228,974.1	69.3	67,880	906,054	260,439
	2017	331,510.1	226,503.2	68.3	71,656	892,586	260,345
	2018	334,179.5	227,407.8	68.0	78,932	892,734	267,101
	2019	340,929.3	229,488.8	67.3	80,813	891,836	277,963
E9	2016	15,423.5	10,022.6	65.0	4444	38,239	12,321
	2017	15,739.9	9956.0	63.3	4802	38,702	12,722
	2018	15,600.3	92,80.5	59.5	5563	36,884	12,325
	2019	16,585.5	10,124.9	61.0	5380	43,563	12,894
Q1	2016	21,275.5	11,844.6	55.7	4520	56,344	21,438
	2017	21,312.2	12,139.1	57.0	4378	55,738	20,605
	2018	20,799.6	11,781.6	56.6	4664	54,882	20,406
	2019	20,875.5	11,736.3	56.2	4899	53,637	20,511
Q2	2016	22,331.9	12,352.5	55.3	4838	59,648	22,287
	2017	22,336.7	12,704.7	56.9	4671	58,994	21,496
	2018	21,764.6	12,351.3	56.7	4998	58,321	21,186
	2019	21,751.6	12,196.0	56.1	5237	56,232	21,318
Q3	2016	3915.3	2054.6	52.5	1044	8839	3756
	2017	3953.1	2041.5	51.6	1150	8712	3417
	2018	3934.6	1769.5	45.0	1167	7760	3813
	2019	4232.0	1930.8	45.6	1293	8506	3994
Q4	2016	11.0	9.7	88.6	0	35	9
	2017	4.1	4.7	112.6	0	17	2
	2018	4.3	3.8	88.2	0	12	3
	2019	4.3	4.6	106.9	0	17	2
Q5	2016	1.7	2.6	146.5	0	10	1
	2017	2.0	2.6	131.6	0	9	1
	2018	1.9	2.7	142.5	0	10	1
	2019	2.6	3.8	146.8	0	14	1
Q6	2016	18.9	20.0	106.1	0	80	13
	2017	15.7	16.2	103.0	0	53	10
	2018	18.3	18.2	99.6	0	72	12
	2019	15.9	15.2	95.6	0	60	10
Q7	2016	8.4	10.1	120.9	0	40	5
	2017	13.1	16.3	124.1	0	70	5
	2018	13.4	15.6	116.3	0	59	7
	2019	13.7	15.8	115.7	0	55	9
A1	2016	126.4	59.7	47.2	29	301	117
	2017	128.0	68.3	53.4	26	336	111
	2018	124.5	75.5	60.6	25	326	103
	2019	127.2	81.3	63.9	39	302	103
A2	2016	9114.0	5780.4	63.4	450	19,285	9051
	2017	9857.6	6243.5	63.3	484	24,897	9375
	2018	10,185.3	7344.9	72.1	590	32,875	9338
	2019	11,153.3	7928.8	71.1	1251	34,787	9455
A3	2016	4246.5	3328.9	78.4	493	12,027	3043
	2017	4276.3	4097.4	95.8	511	16,520	2773
	2018	4026.3	3841.6	95.4	533	14,859	2403
	2019	4104.3	3984.8	97.1	593	14,650	2486

**Table 6:** Results obtained for model A

Group	DMU	2016			2017			2018			2019		
		VRS	CRS	Scale	VRS	CRS	Scale	VRS	CRS	Scale	VRS	CRS	Scale
Group B	CHMA	0.727	0.680	0.936	0.810	0.784	0.967	0.779	0.698	0.896	0.922	0.774	0.839
Group B	CHPVVC	0.925	0.917	0.991	1.000	1.000	1.000	1.000	1.000	1.000	0.987	0.987	1.000
Group B	HDFF	1.000	0.950	0.950	1.000	0.972	0.972	0.906	0.839	0.926	0.972	0.880	0.905
Group B	HSMM	1.000	0.873	0.873	1.000	0.924	0.924	1.000	0.964	0.964	1.000	0.946	0.946
Group C	CHBM	0.656	0.606	0.923	0.625	0.594	0.951	0.769	0.592	0.770	0.891	0.669	0.750
Group C	CHL	0.995	0.871	0.876	0.961	0.774	0.805	0.942	0.704	0.748	0.973	0.788	0.810
Group C	CHS	0.810	0.741	0.915	0.757	0.674	0.890	0.833	0.696	0.836	0.805	0.664	0.825
Group C	CHBV	0.894	0.808	0.904	0.904	0.803	0.887	0.939	0.800	0.852	0.882	0.772	0.875
Group C	CHEDV	0.986	0.848	0.860	0.840	0.792	0.943	1.000	0.986	0.986	0.785	0.688	0.875
Group C	CHMT	0.820	0.762	0.929	0.713	0.643	0.902	0.887	0.755	0.852	0.733	0.624	0.851
Group C	CHTS	1.000	0.881	0.881	1.000	1.000	1.000	0.980	0.874	0.891	1.000	0.894	0.894
Group C	CHUCB	0.744	0.707	0.949	0.802	0.742	0.925	0.787	0.728	0.924	0.662	0.644	0.972
Group C	HSOG	0.891	0.734	0.824	0.785	0.740	0.942	0.742	0.620	0.836	1.000	0.695	0.695
Group C	HDS	0.705	0.684	0.971	0.711	0.654	0.919	0.868	0.708	0.816	0.981	0.705	0.719
Group D	CHTV	0.759	0.581	0.766	0.673	0.526	0.782	0.786	0.541	0.689	0.887	0.568	0.641
Group D	CHTMAD	0.883	0.775	0.878	0.963	0.788	0.819	0.877	0.699	0.796	0.946	0.691	0.731
Group D	CHUA	0.597	0.450	0.754	0.642	0.464	0.723	0.619	0.462	0.746	0.719	0.482	0.670
Group D	CHVNGE	0.946	0.726	0.768	0.916	0.684	0.746	0.916	0.634	0.693	0.912	0.674	0.739
Group D	HESE	0.777	0.761	0.979	0.756	0.718	0.950	0.638	0.597	0.936	0.898	0.787	0.877
Group D	HFF	0.874	0.660	0.755	0.864	0.726	0.840	0.960	0.745	0.776	0.995	0.818	0.823
Group D	HGO	0.821	0.686	0.836	0.748	0.566	0.756	0.796	0.598	0.752	0.702	0.514	0.732
Group E	CHLO	0.813	0.461	0.567	0.538	0.416	0.773	0.683	0.445	0.651	0.516	0.402	0.779
Group E	CHUC	1.000	0.480	0.480	1.000	0.475	0.475	1.000	0.463	0.463	1.000	0.468	0.468
Group E	CHULC	0.983	0.533	0.542	0.974	0.490	0.503	0.943	0.422	0.447	0.717	0.444	0.620
Group E	CHUSJ	0.913	0.753	0.825	1.000	0.772	0.772	1.000	0.773	0.773	1.000	0.731	0.731
Group E	CHUP	0.848	0.542	0.640	0.917	0.511	0.557	1.000	0.663	0.663	0.936	0.726	0.775
Group E	CHULN	0.867	0.687	0.793	0.865	0.489	0.565	0.873	0.478	0.548	0.863	0.505	0.585

Values for VRS. CRS and Scale Efficiency for each year considered

**Table 7:** Relevant literature summary

Authors	Variables			Conclusions
	Inputs	Outputs	Other	
Campanella et al. (2017)	Number of Beds;Number of Doctors;Number of Nurses(per patient)	30-day risk-adjusted mortality	Contextual factors$^\mathrm{{a}}$ such as location,demographic information and economic situation	A better efficiency could be achieved without penalise quality
Chang et al. (2011)	Number of Beds; Number of Doctors; Number of Nurses; Number of supporting personnel	Outpatients Treated; Number of Surgeries; Number of patient days; Number of net inpatient mortality	-	Is possible for hospitals to improve their operating efficiency without sacrificing quality
Chatfield (2014)	Number of Beds; Operation Costs without depreciation and labour expenses; Total employees; Service mix	Inpatients Treated; Outpatients Treated; Quality (composite measure calculated using 20 quality variables)	-	Quality outcomes are not penalised by a higher efficiency. A trade-off between efficiency and quality was not observed
De Nicola et al. (2014)	Number of Beds; Number of Doctors; Number of Nurses	Number of total patients; Case mix	Environmental variables$^\mathrm{{b}}$ such as organisational models reimbursement systems location, year and population	Healthcare efficiency increases if a quality service is provided
Ferreira et al. (2020)	Staff costs; Operation Costs; Costs of goods sold and consumed; Costs with outsourcing; Hospital days	Inpatients Treated; Emergency cases; First medical appointments; Follow-up scheduled medical appointments; Outpatient surgeries; Conventional surgeries; Urgent surgeries; Births; Hospital days	Environmental variables$^\mathrm{{c}}$ such as mortality rate, population demographics, birth rate and illiteracy rate	There is no trade-off between efficiency, quality and access. Efficiency can be improved without decreasing quality and access
Gao and Wang (2020)	Number of Beds; Number of Doctors; Number of Nurses; Number of supporting staff	Outpatients Treated; Inpatients Treated	Environmental variables$^\mathrm{{b}}$ such as population average age, location and hospital classification	Higher efficiency scores imply a better equity regarding access
Gok and Sezen (2013)	Number of Beds; Number of specialist physicians; Number of non-specialist physicians	Bed utilisation rate; Bed turnover rate; Number of Surgeries; Number of births; Outpatients Treated; Average facility inpatient days; Inpatients Treated	Hospital size$^\mathrm{{b}}$; Average length of stay$^\mathrm{{b}}$; Structural quality$^\mathrm{{b}}$	The relationship between quality and efficiency is dependent on the hospital size. In small and medium hospitals there is a trade-off between the two, which does not happen in larger hospitals
Gorgemans et al. (2018)	Number of Beds; Number of Doctors; Number of Nurses	Inpatients Treated; Outpatients Treated	Mortality rate$^\mathrm{{d}}$; CVC Bloodstream Infections$^\mathrm{{b}}$; Postoperative PE$^\mathrm{{e}}$ or DVT$^\mathrm{{f}}$ and sepsis	Improvements in efficiency could produce improvements in quality, although the relation is not valid for all cases
Khushalani and Ozcan (2017)	Number of Beds; Non-physician FTE$^\mathrm{{g}}$; Operation Costs; Resident Nurses to Total Nursing FTE ratio; Hi-tech service mix	Outpatients Treated; % of patients who would definitely recommend the hospital; % of patients who gave the hospital a high rating	Net patient revenue per bed$^\mathrm{{h}}$; Mortality$^\mathrm{{h}}$; Readmission rates$^\mathrm{{h}}$; Case-mix adjusted discharges$^\mathrm{{i}}$; Number of Surgeries$^\mathrm{{i}}$; Emergency Visits$^\mathrm{{i}}$	There is no trade-off between efficiency and quality
Kittelsen et al. (2015)	Hospital Costs	Medical inpatients; Surgical inpatients; Outpatients Treated	Readmission$^\mathrm{{b}}$; Mortality$^\mathrm{{b}}$; Patient Satisfaction$^\mathrm{{b}}$	A clear trade-off between costs and quality was not detected, although a significant trade-off between productivity and readmissions rates was observed
Mitropoulos (2021)	Number of Beds; Number of Doctors; Expenditure	Quality of care; Patient safety	Inpatients Treated$^\mathrm{{i}}$; Outpatients Treated$^\mathrm{{i}}$; Unmet Demand$^\mathrm{{i}}$	A trade-off between production and service quality was detected, suggesting that improving efficiency might worsen quality
Onder et al. (2021)	Number of Beds; Number of Employees; Inpatients Treated	Readmission Rate; Operation Costs	Environmental variables$^\mathrm{{j}}$ such as wages index, location and teaching status	Improvements in quality results in a better efficiency
van Ineveld et al. (2016)	FTE employed by the hospital (except physicians); Number of Doctors; Operation Costs	Inpatients Treated; Outpatients Treated; Number of day care treatments	Number of Decubitus Ulcers$^\mathrm{{c}}$	Quality and economic improvements evolve in the same way
Varabyova et al. (2017)	Number of Doctors; Number of Nurses	Inpatients Treated	Patient satisfaction$^\mathrm{{k}}$ mortality rate and radiation exposure	There is no clear trade-off between efficiency and quality. The impact of quality in efficiency depends on the measure used

$^\mathrm{{a}}$ Used in a Tobit regression model

$^\mathrm{{b}}$ Used in a regression model

$^\mathrm{{c}}$ Used as external non-discretionary variables

$^\mathrm{{d}}$ Used in a logistic regression

$$^\mathrm{{e}}$$ Pulmonary Embolism

$^\mathrm{{f}}$ Deep Vein Thrombosis

$^\mathrm{{g}}$ Full Time Equivalent

$^\mathrm{{h}}$ Used as a carry over

$^\mathrm{{i}}$ Used as a link

$^\mathrm{{j}}$ Used in a truncated regression

$^\mathrm{{k}}$ Used in a non-parametric regression

## Footnotes

1 https://benchmarking-acss.min-saude.pt/.

2 https://www.sns.gov.pt/transparencia/.

3 Result charts can be found at: https://drive.google.com/drive/folders/1ZTPpJjmw5_iFOvsjMPeUimwOFaTjAHZj?usp=sharing.
